# Totally laparoscopic anterior resection with transvaginal assistance and transvaginal specimen extraction: a technique for natural orifice surgery combined with reduced-port surgery

**DOI:** 10.1007/s00464-013-3120-3

**Published:** 2013-08-16

**Authors:** Atsushi Nishimura, Mikako Kawahara, Keisuke Honda, Takahiro Ootani, Tomoyuki Kakuta, Chie Kitami, Shigeto Makino, Yasuyuki Kawachi, Keiya Nikkuni

**Affiliations:** 1 Department of Surgery, Institute of Gastroenterology, Nagaoka Chuo General Hospital, 2041 Kawasaki-cho, Nagaoka, Niigata 940-8653 Japan; 2 Department of Obstetrics and Gynecology, Nagaoka Chuo General Hospital, Nagaoka, Niigata Japan

**Keywords:** Laparoscopic colectomy, Natural orifice specimen extraction (NOSE), Transvaginal specimen extraction, Reduced-port surgery

## Abstract

**Background:**

Natural orifice specimen extraction (NOSE) has been developed as a means of decreasing the incidence of surgical wound complications. However, NOSE performed using a conventional multiport technique has been reported previously. The current authors performed totally laparoscopic anterior resection with transvaginal specimen extraction (TVSE) using the reduced-port surgery (RPS) technique. The Alexis wound retractor (Applied Medical, Rancho Santa Margarita, CA, USA) and Free Access (Top Corporation, Tokyo, Japan) were attached to the transvaginal route for transvaginal assistance and smooth specimen extraction. The authors documented this simple and safe technique and its short-term results.

**Methods:**

Data were prospectively collected for five patients who underwent totally laparoscopic anterior resection with TVSE for colorectal cancer between June 2012 and December 2012. A multiport access device (GelPOINT advanced-access platform; Applied Medical) was inserted into the navel, and a 5-mm port was inserted into the right lower quadrant to be used as a drain site. Transverse transvaginal posterior colpotomy then was performed. One ring of an Alexis ring pair was inserted into the peritoneal cavity through the vagina. The other white ring was placed outside of the vagina and then covered with a Free Access to maintain the pneumoperitoneum for insertion of a 12-mm port. Lymph node dissection and transection of the distal colon were performed with transvaginal assistance. The specimen then was extracted transvaginally. After the Alexis had been removed, the vaginal incision was closed transvaginally. End-to-end colorectal anastomosis was performed using the double-stapling technique.

**Results:**

Transvaginal extraction was completed in all five cases. The median operation time was 235 min. One case was complicated by chyloperitoneum. The median hospital stay was 6 days. Only one patient required intravenous analgesics once on postoperative day 1. All the patients remained disease free.

**Conclusion:**

Totally laparoscopic anterior resection using TVSE with RPS appears to be feasible, safe, and oncologically acceptable for selected cases.

Rapid advances in laparoscopically assisted colectomy (LAC) have reduced the invasiveness of the procedure. Conventional techniques for LAC require an abdominal minilaparotomy for extraction of the specimen. However, the incision often causes postoperative pain, wound infection, and incisional hernia, which reduce the advantages of LAC [1–3]. Natural orifice specimen extraction (NOSE), which does not involve an extraction minilaparotomy, has been developed as a means of decreasing the incidence of surgical wound complications.

We previously performed totally laparoscopic sigmoid colectomy and anterior resection for colon cancer using transanal specimen extraction (TASE) [4, 5]. However, some limitations have been associated with this procedure. The TASE procedure may not be possible in patients with bulky tumors, a thick mesentery, a narrow rectum, or anal stenosis. In addition, the risk of intracorporeal contamination by tumor cells or bacteria and damage to the function of the anal sphincter have not been fully investigated.

On the other hand, laparoscopic colectomy with transvaginal specimen extraction (TVSE) has been reported previously [1–3, 6–10]. According to the reports, TVSE resulted in reduced wound pain, a shorter hospital stay, and good cosmetic outcomes. No cancer recurrence has been reported after this procedure. We also began performing TVSE for colon cancer in October 2010 and have carried out a procedure combined with reduced-port surgery (RPS) since June 2012. We document this innovative and safe technique and its short-term results.

## Methods

Transvaginal specimen extraction with RPS was indicated for patients who previously underwent vaginal delivery with clinical stage T3 or lower primary tumors located from the sigmoid colon to the upper rectum. We limited the indication to menopausal women only, excluding patients whose tumor covered more than half of the colon circumference and obese patients. Between June 2012 and December 2012, five patients underwent the aforementioned procedure (Table 1), which was performed with institutional review board approval.

**Table 1:** Patient characteristics

Case	Age (years)	BMI (kg/m^2^)	Tumor size (cm)	Stage	Operation	Anvil placement	Operation time (min)	Blood loss (ml)	No. of lymph nodes harvested	POHS	Complications
1	54	21.0	3.7	T3N0	S	IC	300	3	22	4	–
2	81	16.2	3.0	T3N0	LAR	EC	235	40	17	7	–
3	84	18.7	0^a^	T1N0	S	EC	186	5	13	6	–
4	57	27.3	0.8	T1N1	S	EC	255	20	19	5	–
5	61	23.4	2.2	TisN0	S	EC	201	5	13	11	Chyloperitoneum

*BMI* body mass index, *POHS* postoperative hospital stay, *S* sigmoidectomy, *IC* intracorporeal, *LAR* low anterior resection, *EC* extracorporeal

^a^Case managed by endoscopic mucosal resection

### Technique

The patient was positioned in the supine/sprit-leg position, with the two legs on a lithotomy positioning device so the patient could be placed in the lithotomy position to allow adequate exposure for the transvaginal procedure. The abdomen, perineum, and vagina were prepared antiseptically.

**Fig. 1:**
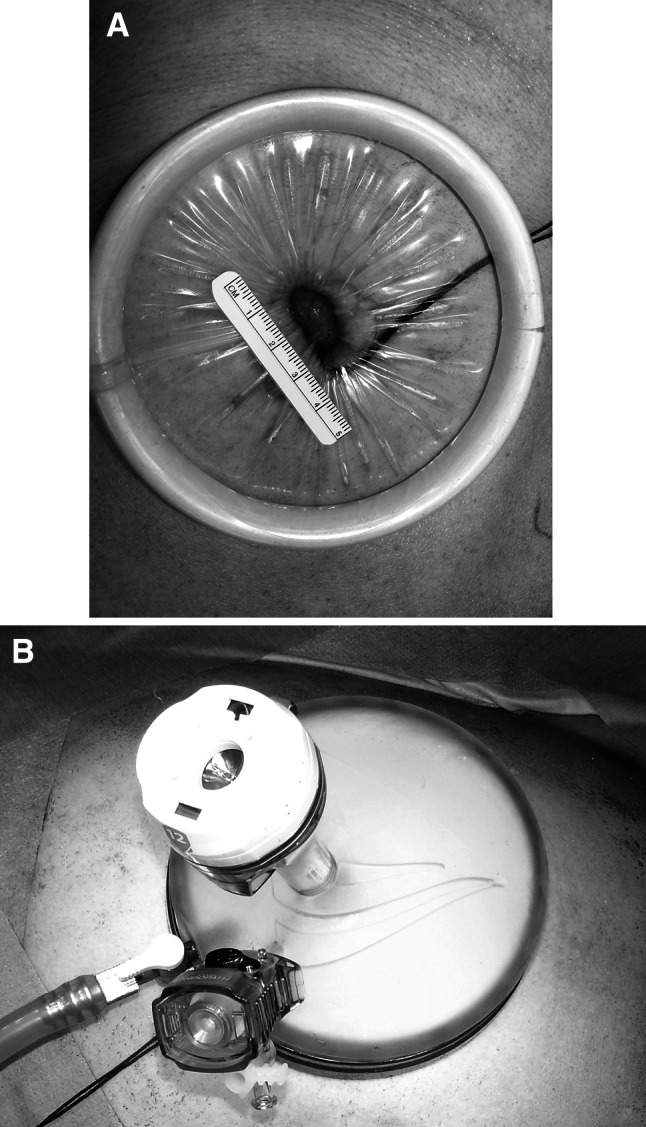
**A** A GelPOINT advanced-access platform placed through a 2-cm-long minilaparotomy in the navel. **B** Placement of 12- and 3-mm ports in the GelPOINT

**Fig. 2:**
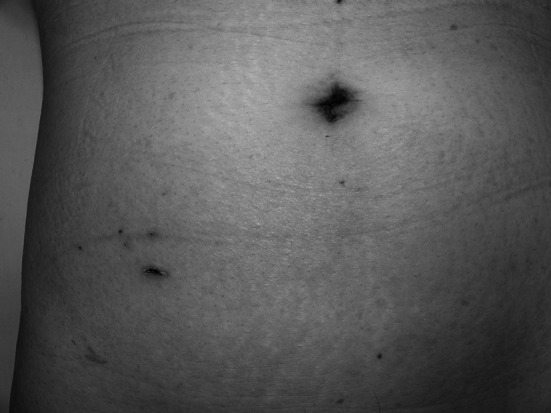
Abdomen of the patient showing the port incision scars 2 weeks after surgery

The multiport access device (GelPOINT advanced-access platform; Applied Medical, Rancho Santa Margarita, CA, USA) was placed through a 2-cm-long minilaparotomy in the navel (Fig. 1A), and the abdomen then was insufflated to 10 mmHg. A 12-mm port for a laparoscope or linear stapling device and a 3-mm port for the surgeon’s left hand were placed in the GelPOINT (Fig. 1B). The third port was a 5-mm surgeon’s operating port in the right lower quadrant, which was used as a drain site (Fig. 2).

**Fig. 3:**
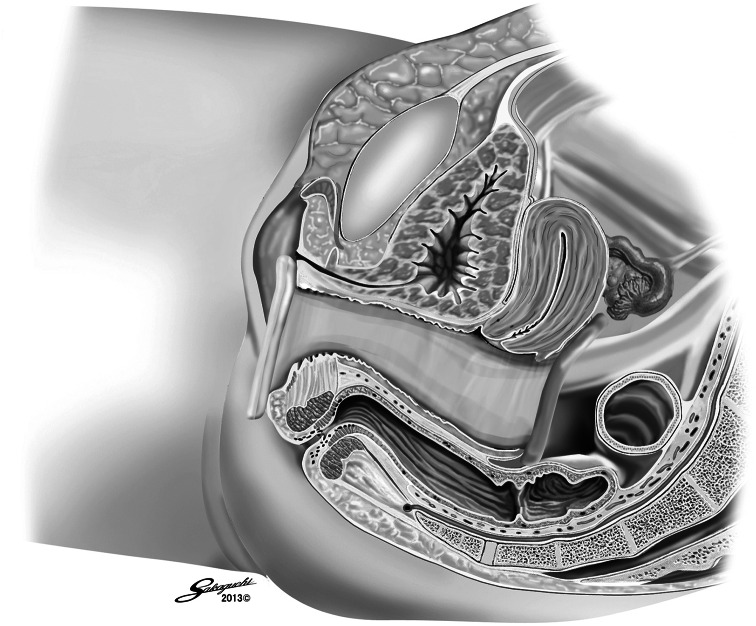
One ring of an Alexis ring pair inserted into the abdominal cavity transvaginally through the colpotomy. The other white ring was placed outside the vagina

**Fig. 4:**
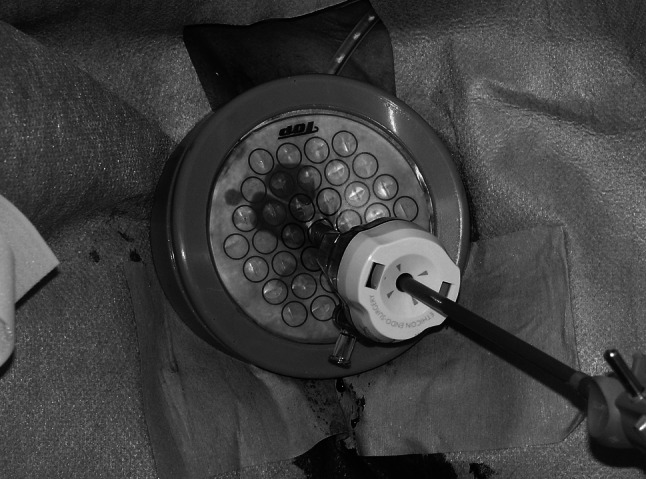
White ring of the Alexis covered with a Free Access to maintain the pneumoperitoneum for insertion of a 12-mm port to be used by the assistant

Next, the patient was placed in the lithotomy position. With the help of the gynecologist, a 2- to 3-cm-long transverse transvaginal posterior colpotomy was performed under laparoscopic guidance. One S-sized Alexis wound retractor ring belonging to a ring pair (Applied Medical, Rancho Santa Margarita, CA, USA) was carefully inserted into the abdominal cavity transvaginally through the colpotomy. The other white ring was placed outside the vagina (Fig. 3). The cylindrical membrane of the Alexis was rolled around the white ring to expand the orifice of the colpotomy gently. The white ring of the Alexis then was covered with a Free Access (Top Corporation, Tokyo, Japan) to maintain the pneumoperitoneum for insertion of a 12-mm port to be used by the assistant (Fig. 4). The surgeon and scope operator were positioned on the patient’s right side, with the assistant sitting between the legs.

The inferior mesenteric artery was ligated at its point of origin from the aorta before the tumor was mobilized. All sigmoidal branches were removed with the specimen. The sigmoid, the distal part of the descending colon, and the rectum were mobilized. No case required splenic flexure mobilization.

**Fig. 5:**
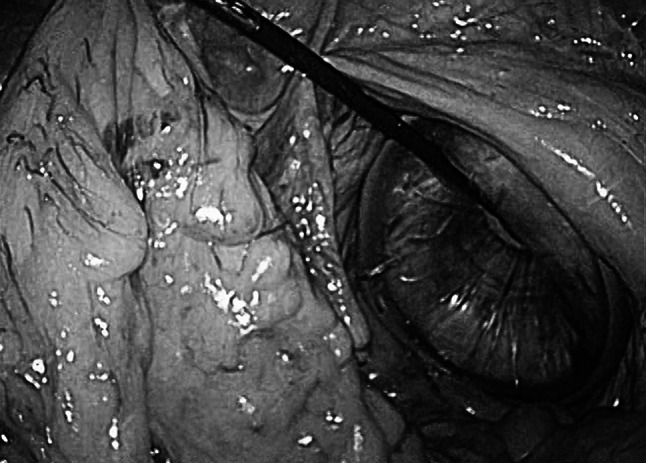
Sigmoid colon retracted by laparoscopic forceps inserted through the vagina

Laparoscopic forceps were inserted transvaginally and used to retract the mesocolon (Fig. 5), mesorectum, and pedicle of the inferior mesenteric artery to aid in the dissection as well as in the insertion and removal of laparoscopic gauze. We then inserted a laparoscope through the 5-mm port in the right lower quadrant. The section of the rectum distal to the tumor was clamped with a detachable clip inserted through the 12-mm port in the GelPOINT.

**Fig. 6:**
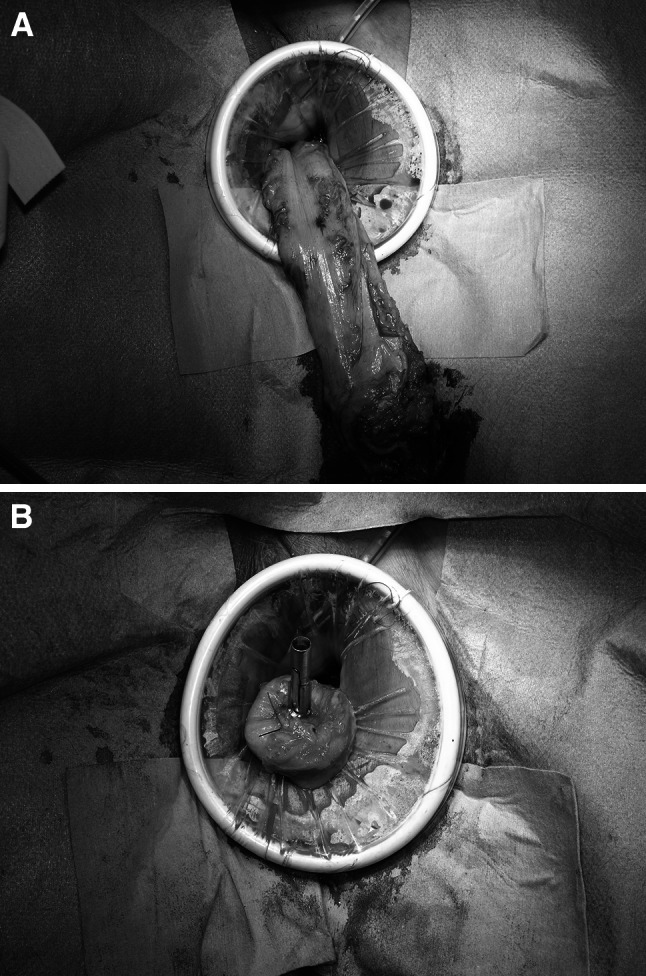
**A** Transvaginal specimen extraction. **B** Anvil head of the circular stapling device inserted into the proximal colon through the vagina

After irrigation of the remnant rectum with 1 l of diluted povidone–iodine solution, the rectum was transected with a linear stapling device (Echelon 60; Ethicon Endo-Surgery, Cincinnati, OH, USA). Transection of the proximal colon was performed through the vagina in four cases. A Babcock was inserted through the Alexis, and the edge of the proximal colon was grasped and extracted transvaginally (Fig. 6A).

After measurement of an appropriate distance from the tumor, the proximal colon was transected. The anvil head of the circular stapling device (CDH 29; Ethicon Endo-Surgery, Cincinnati, OH, USA) was inserted into the proximal colon with 2/0 Prolene purse-string sutures (Fig. 6B). After placement of the proximal colon back into the abdominal cavity, the Alexis was removed transvaginally.

For a case with tumor located near the descending colon, transection of the proximal colon and insertion of the anvil head were performed intracorporeally. The vaginal incision then was closed with double-layer running absorbable sutures transvaginally.

Next, end-to-end colorectal anastomosis was performed with a circular stapler using the double-stapling technique. After the pelvic cavity had been irrigated with 1 l of saline, a pelvic drain was inserted through the incision in the right lower quadrant.

## Results

For five patients, TVSE with RPS was attempted and completed successfully (Table 1). No intraoperative vaginal injuries were encountered. The median operation time was 235 min (range 186–300 min), and the median blood loss was 5 ml (range 3–40 ml). The median number of harvested lymph nodes was 17 (range 13–22).

One case was complicated by chyloperitoneum leaking from a drain (63–136 ml/day). However, this decreased spontaneously, and the drain was removed on postoperative day (POD) 7. The patient was discharged on POD 11. Four other patients left our hospital according to our clinical path for laparoscopic colectomy without any negative variance.

The median hospital stay was 6 days (range 4–11 days). Four patients were able to walk on POD 1, and one was able to walk on POD 2. Flatus was passed by four patients on POD 1 and one patient on POD 2. The epidural catheter was removed on POD 1.

Pain was rated by the patients on a validated numeric rating scale (NRS) ranging from 0 (no pain) to 10 (worst pain imaginable). The median NRS score on PODs 1, 2, 3, 4, and 5 were respectively 2, 3, 2, 1, and 1. Only one patient required intravenous analgesics once on POD 1. No other patient required any analgesics after removal of the epidural catheter.

The follow-up period ranged from 2 to 9 months. One node-positive patient underwent postoperative chemotherapy for 6 months. Follow-up examinations were scheduled at 2 weeks, then at 1, 2, 3, 6, 9, and 12 months, and finally every 6 months thereafter until 5 years. Plans were made for all the patients to undergo computed tomography of the chest, abdomen, and pelvis every 6 months. There was no evidence of metastasis.

## Discussion

Laparoscopic surgery is progressing toward RPS to achieve improved short-term patient outcomes. Recently, an investigation of natural orifice transluminal endoscopic surgery (NOTES) has proceeded with the aim of further reducing the procedure’s invasiveness [11]. However, numerous technical issues need to be resolved before its universal application to malignant diseases.

Natural orifice specimen extraction has been proposed as a bridging technique to NOTES. Breda et al. [12] first performed transvaginal NOSE for a patient with a small, nonfunctional tuberculous kidney.

The vagina has been established as one of the preferred routes for specimen extraction in NOSE because of its improved healing and elasticity [2, 3, 7]. Many studies have reported laparoscopic colectomy with TVSE [1–3, 6–10]. However, previously reported TVSE procedures have been performed with a conventional multiport technique, which is considered unsatisfactory from the viewpoint reduced invasiveness and improved cosmetic outcomes. We attempted to perform the procedure combined with the RPS technique and obtained good results.

Only a few studies have evaluated the reduced invasiveness of laparoscopic colectomy with TVSE. Park et al. [9] reported a case–control study that compared the clinical outcomes of totally laparoscopic hemicolectomy with TVSE and the conventional laparoscopically assisted approach for right-sided colonic cancer. After TVSE, the patients had less pain on POD 1 (4.2 vs. 5.7 on VAS; *P* = 0.001) and POD 3 (2.6 vs. 3.5 on VAS; *P* = 0.010), as well as a shorter hospital stay (7.9 vs. 8.8 days; *P* = 0.003).

We adopted the RPS technique to obtain further relief from pain and good cosmetic outcomes. The GelPOINT consists of an abdominal wound protector and a peculiar gel seal cap applied to the protector. We can insert any number of ports in the cap and remove them. The greatest advantage of this instrument is the flexibility and mobility of the port position in the gel seal cap without the escape of carbon dioxide (CO_2_) gas.

The GelPOINT is applicable for incisions 1.5–7 cm long. We set up the length of the major axis of minilaparotomy at the navel expanded by GelPOINT as <2 cm. It is important to distinguish our procedure from single-port surgery due to the size of the largest incision. Our minilaparotomy is too small for the extraction of colon specimens or insertion of the anvil head. The closed navel wound is invisible, and the only visible scar is a 5-mm scar in the right lower quadrant.

In this study, our patients required fewer analgesics postoperatively than previously reported cases managed by TVSE with the multiport technique [7, 9]. We believe our procedure acquired reduced invasiveness equal to hybrid NOTES and better cosmetic outcomes than NOSE with the multiport technique. However, further prospective investigations are necessary to establish its superiority over the conventional laparoscopically assisted approach and NOSE with the multiport technique in terms of inclusive quality of life.

Reduced-port surgery has several disadvantages over multiport laparoscopic surgery such as the clashing of instruments, the lack of tissue triangulation, and inadequate exposure [13–15]. To overcome these disadvantages, we adapted the Alexis and Free Access to the vagina for transvaginal assistance.

The Alexis, a polyurethane wound retractor manufactured by Applied Medical, was initially developed to protect against abdominal wounds [16]. It has a flexible cylindrical membrane attached to two semi-rigid rings on each end.

We initially used the Alexis to protect the rectum at TASE [4, 5]. Kho et al. [17] used the Alexis to extract large uteri through the vagina. Free Access is designed to attach to the white ring of the Alexis for maintenance of an airtight condition and for insertion of some ports. Because it was so easy to retract the mesocolon, mesorectum, and the pedicle of the inferior mesenteric artery transvaginally, we could maintain optimal tissue triangulation and exposure. Moreover, we could dissect the adhesion between the omentum and former laparotomy scar transvaginally using two ports for forceps and an energy device simultaneously inserted to the Free Access.

There was no necessity to mobilize the splenic flexure in our series. It appears to be a technical issue with our procedure, but it is possible if we use detachable organ retraction device such as the EndoGrab retractor (Virtual Ports, Ltd., Misgav, Israel). We must not hesitate to place an additional port besides the umbilical region for cases involving difficulty in its mobilization. Hereafter, we need better adapted instruments, such as more flexible long forceps and energy devices, to avoid interruption by the promontory of the sacrum for cases in which we attempt lymph node dissection and mobilization of the left colon transvaginally. The complication of chyloperitoneum occurred in one of our cases in which we were able to complete TVSE without any difficulty. We believe that this complication may have been caused by insufficient sealing of the lymphatic vessels around the inferior mesenteric artery and that it was unrelated to our use of the new technique.

It is of utmost importance that oncologic safety be secured in TVSE for colorectal cancer. No vaginal metastasis has been reported in 67 colorectal cancer cases [10]. However, there is a potential risk of cancer cell exfoliation, implantation, and local recurrence in the abdominal cavity or vaginal stump. Some investigators have recommended the use of a protective barrier or a specimen bag to reduce the incidence of these problems [1–3]. McKenzie et al. [2] reported that the risk of tumor seeding during transvaginal delivery was no higher than that associated with transabdominal extraction providing proper oncologic principles are followed and specimen handling is performed using a specimen retrieval bag.

We believe it is important for the specimen to take a “linear” form to facilitate its extraction through the vagina. Accordingly, we attempted to protect the vagina by using the Alexis retractor instead of a specimen bag.

It also is important to establish safety with colpotomy and transvaginal access.

Ghezzi et al. [18] reviewed 23 studies with a total of 501 patients and found only one complication (severe vaginal bleeding) (0.2 %) directly attributable to the colpotomy. A systematic review by Diana et al. [10] reported that the rate of severe complications was 3.7 % for a left-sided colectomy and 2 % for a right-sided colectomy. Two significant complications were pelvic seroma and rectovaginal fistula.

We believe that transvaginal posterior colpotomy under laparoscopic guidance is very safe from injury to other organs. There may be a potential risk of injury to the vagina during specimen extraction. We believe that the Alexis prevents excessive pressure to the vagina, resulting in minimal risk of it tearing during the extraction of bulky specimens. We must switch to conventional LAC for cases involving strong resistance to the passage of the specimen due to a size mismatch between the vagina and the specimen.

The impact of transvaginal access on postoperative sexual function, dyspareunia, and pregnancy rate has not been fully investigated. Tarantino et al. [8] reported on 34 cases of transvaginal rigid-hybrid NOTES anterior resection for diverticular disease, showing that at 6 weeks postoperatively, sexual function did not differ significantly from the preoperative status. Of 63 patients who underwent vaginal extraction of benign gynecologic masses, 51 resumed sexual activity, and none reported dyspareunia [18]. Paraiso et al. [19] evaluated postoperative sexual function using a validated questionnaire and reported a high rate of dyspareunia relative to that of other studies. This finding may suggest difficulties in researching sexual function and the fact that the results of research depend on the method used in the evaluation. Although we indicated TVSE for menopausal females only, this procedure could be performed for premenopausal patients. Further prospective investigations using appropriate validated scales are necessary.

This procedure has some limitations. The TVSE procedure may not be possible for patients with bulky tumors, previous pelvic surgery or radiation, or a narrow vagina. In addition, further prospective investigations are necessary to establish the indications for this procedure, and the risk of damaging sexual function has not been investigated. Therefore, a randomized control study should be performed.

In conclusion, we believe that totally laparoscopic sigmoid colectomy and anterior resection using TVSE with the RPS technique is feasible, safe, and oncologically acceptable for selected cases. Further studies are necessary to establish whether this procedure is an appropriate option for the laparoscopic management of tumors located from the sigmoid colon to the upper rectum.
